# Pharmacokinetics, Safety, and Efficacy of an Allometric Miltefosine Regimen for the Treatment of Visceral Leishmaniasis in Eastern African Children: An Open-label, Phase II Clinical Trial

**DOI:** 10.1093/cid/ciy747

**Published:** 2018-09-05

**Authors:** Jane Mbui, Joseph Olobo, Raymond Omollo, Alexandra Solomos, Anke E Kip, George Kirigi, Patrick Sagaki, Robert Kimutai, Lilian Were, Truphosa Omollo, Thaddaeus W Egondi, Monique Wasunna, Jorge Alvar, Thomas P C Dorlo, Fabiana Alves

**Affiliations:** 1 Centre for Clinical Research, Kenya Medical Research Institute, Nairobi; 2 Department of Medical Microbiology, Leishmaniasis Unit, College of Health Sciences, Makerere University, Kampala, Uganda; 3 Drugs for Neglected Diseases Initiative, Nairobi, Kenya; 4 Drugs for Neglected Diseases Initiative, Geneva, Switzerland; 5 Department of Pharmacy & Pharmacology, Antoni van Leeuwenhoek Hospital/the Netherlands Cancer Institute, Amsterdam; 6 Amudat Hospital, Uganda

**Keywords:** visceral leishmaniasis, Eastern African children, miltefosine, allometric regimen, drug pharmacokinetics

## Abstract

**Background:**

Convenient, safe, and effective treatments for visceral leishmaniasis in Eastern African children are lacking. Miltefosine, the only oral treatment, failed to achieve adequate efficacy, particularly in children, in whom linear dosing (2.5 mg/kg/day for 28 days) resulted in a 59% cure rate, with lower systemic exposure than in adults.

**Methods:**

We conducted a Phase II trial in 30 children with visceral leishmaniasis, aged 4–12 years, to test whether 28 days of allometric miltefosine dosing safely achieves a higher systemic exposure than linear dosing.

**Results:**

Miltefosine accumulated during treatment. Median areas under the concentration time curve from days 0–210 and plasma maximum concentration values were slightly higher than those reported previously for children on linear dosing, but not dose-proportionally. Miltefosine exposure at the start of treatment was increased, with higher median plasma concentrations on day 7 (5.88 versus 2.67 μg/mL). Concentration-time curves were less variable, avoiding the low levels of exposure observed with linear dosing. The 210-day cure rate was 90% (95% confidence interval, 73–98%), similar to that previously described in adults. There were 19 treatment-related adverse events (AEs), but none caused treatment discontinuation. There were 2 serious AEs: both were unrelated to treatment and both patients were fully recovered.

**Conclusions:**

Allometric miltefosine dosing achieved increased and less-variable exposure than linear dosing, though not reaching the expected exposure levels. The new dosing regimen safely increased the efficacy of miltefosine for Eastern African children with visceral leishmaniasis. Further development of miltefosine should adopt allometric dosing in pediatric patients.

**Clinical Trials Registration:**

NCT02431143.

## Teaser

Allometric miltefosine dosing for 28 days in Eastern African children with visceral leishmaniasis demonstrated increased and less-variable exposure than linear miltefosine dosing, with improved efficacy and a satisfactory safety profile.

Visceral leishmaniasis (VL; also known as kala-azar) is the most lethal form of leishmaniasis, inevitably requiring treatment to prevent mortality [1]. Around 200000–400000 new cases of VL occur worldwide each year (data from 2004–2008 [2]). Cases of VL have sharply decreased in South Asia following an elimination campaign [3, 4], leaving the Eastern Africa region with the highest burden worldwide, with 29400 to 56600 cases estimated annually [2].

A limited number of drugs are available for VL treatment [5] and all of them have limitations related to either toxicity, parenteral administration, cost, and/or a requirement of cold chain. In Eastern Africa, the World Health Organization recommended 17 days of combination treatment of sodium stibogluconate (20 mg/kg/day Sb^5+^) and paromomycin (11 mg base/kg/day) [5]. This combination has an efficacy rate of 91.4% [6], but requires 17 days of 2 injections, and antimonial is associated with infrequent but significant life-threatening toxicity (cardio-toxicity, acute pancreatitis) [5].

Miltefosine, an alkylphosphocholine analogue initially developed as an anti-cancer drug, has reemerged as the only effective oral VL treatment [7]. In initial trials performed in India, miltefosine at 2.5 mg/kg/day for 28 days had high efficacy rates (>90% at 6 months follow-up) in patients aged ≥12 years [8] and in children aged 2–11 [9]. In Ethiopian adult VL human immunodeficiency virus–negative male patients (aged ≥15 years), miltefosine (100 mg/day for 28 days) achieved a 6-month cure rate of 75.6% (99/131), although 19.1% of participants were lost to follow-up. The efficacy for those who completed the 6-month follow-up was 93.4%, with a 5.7% relapse rate and 1 case of death [10]. A phase II trial (LEAP 0208; NCT01067443) [11] in Sudan and Kenya comparing miltefosine alone (2.5 mg/kg/day for 28 days) with miltefosine (2.5 mg/kg/day for 10 days) in combination with liposomal amphotericin B (10 mg/kg single dose) achieved 6-month cure rates of 72% and 77%, respectively. Although this trial produced discouraging efficacy data, it provided important pharmacokinetic data for miltefosine monotherapy in the Eastern African population, showing lower systemic miltefosine exposure in children (<12 years) than in adults with conventional linear mg/kg miltefosine dosing [11]. There was a corresponding lower cure rate in these children; cure rates after 6 months of treatment were 59% in children and 86% in adults (*P* = .05). The lower efficacy of miltefosine monotherapy in pediatric VL was also evident in previous studies in Nepal and India; children with VL had higher rates of failure (6.4% versus 3.4% in adults) [9] and higher relapse risks [12, 13]. Both for Eastern Africa [14] and Asia [13, 15], higher failure rates due to relapses were associated with lower miltefosine exposure [15]. Model-based simulations of an allometric dosing regimen, based on non-linear scaling of the dose in children based on their fat-free mass, predicted a level of miltefosine exposure in children equivalent to that achieved in adults receiving conventional dosing [15]. We tested this prediction in Eastern African children with VL, aiming to increase drug exposure and correspondingly increase the cure rate. Because the required allometric dosing involves oral doses exceeding conventional dosing, we also assessed its safety in children.

## METHODS

### Study Patients

We recruited 30 children with primary VL, aged 4–12 years, at 2 clinical sites: Kacheliba, West Pokot County, Kenya, and Amudat, Karamoja sub-region, Uganda. All patients fulfilled the trial inclusion and exclusion criteria (Supplementary Material). They showed VL clinical signs and symptoms and had a confirmatory parasitological microscopic diagnosis. Their age varied between ≥4 and ≤12 years and they weighed <30 kg. All had primary, non-severe VL (based on clinical and hematological parameters, as per exclusion criteria) and had not received anti-leishmanial drugs within the previous 6 months. None suffered severe malnutrition or any serious underlying disease or concomitant severe infection.

### Study Drug

Miltefosine medication was Impavido in 10 mg and 50 mg capsules (Paladin Labs Inc., Montreal, Canada), in aluminium–aluminium blister foil packs.

### Treatment and Procedures

Patients were hospitalized for screening, baseline procedures, and the 28-day treatment duration, and assessed during outpatient follow-ups on days 56 and 210 after the start of treatment. All patients received 28-day allometric miltefosine dosing twice daily after a meal, and were under observation by the nurse until 30 minutes after each administration to record any vomiting. The allometric dose was determined using the patient’s sex and baseline height and weight, according to tables adapted from Dorlo and colleagues [15] (Supplementary Material). Plasma samples were collected at screening; during treatment at days 1 (8 hours after first dose), 7, 14, 21, and 28 (before miltefosine administration); and at days 56 and 210 during the follow-up visits.

### Trial Design

This was a phase II, open-label clinical trial, registered at the U.S. clinical trial registry (under NCT02431143) and conducted in accordance with the trial protocol, the International Conference on Harmonization guidelines for Good Clinical Practice, local regulations, and the Declaration of Helsinki. Ethical approvals were obtained from institutional ethics committees at the Kenya Medical Research Institute and at Makerere University, Uganda. Individual informed consent was obtained from parents/guardians and assent was obtained from participating patients, when applicable, as per country regulations.

The study objectives were to characterize the pharmacokinetics, safety, and efficacy of a miltefosine allometric regimen given for 28 days in Eastern African children with primary VL. The primary pharmacokinetic endpoints were total drug plasma exposure (area under the concentration time curve [AUC] from days 0 to 210) and plasma maximum concentration (C_max_).

The primary safety endpoints were frequency and severity of adverse events (AEs), serious adverse events (SAEs), and AEs necessitating treatment discontinuation. The secondary efficacy endpoints were cure rates at days 28 and 210 after the start of treatment (cure rate = proportion of patients recovering from clinical signs and symptoms of infection, having a negative microscopic reading for parasitaemia at day 28, and not requiring any rescue treatment up to day 210).

### Plasma Miltefosine Bioanalysis

Samples’ storage and transportation conditions were monitored and maintained maximally at -20°C; no deviations were noted. Plasma miltefosine concentrations were quantified using liquid chromatography coupled to tandem mass spectrometry, as previously published [16]. The performance of the bioanalysis is described in detail in the online Supplementary Material.

### Pharmacokinetic Analyses

The plasma miltefosine data were managed using R (version 3.1.2). A standard 2-stage non-compartmental pharmacokinetic analysis was performed with the R package “ncappc” [17]. Unless indicated otherwise, data are represented as median (range) and statistical tests were performed using a Mann-Whitney U-test.

### Safety Assessment

Treatment safety was assessed at each visit by the routine recording of AEs that occurred since the previous visit; blood sampling for measurements of hematological and clinical chemistry parameters; and assessment of vital signs and physical condition. The laboratory parameters were graded according to Common Terminology Criteria for Adverse Events (CTCAE) v4.0 and clinically-relevant values were recorded.

### Clinical Assessment of Efficacy

The clinical assessment of VL was performed at screening; on days 3, 7, 14, 21, and 28 of treatment; and on follow-up days 56 and 210. This involved measurements of axillary temperature, the size of the spleen and liver, and body weight. VL symptoms were also recorded. Parasitological assessments were performed at baseline and on day 28, using microscopic examinations of spleen aspirate or, under specific circumstances (see Study Protocol in online Supplementary Methods), bone marrow aspirates. A cure at the end of treatment (day 28) was defined as the absence of clinical signs and symptoms of VL (patient afebrile, spleen size reduced, and improvement of symptoms and hematological parameters) and the microscopic absence of parasites from the spleen or bone marrow aspirate. A definitive cure at day 210 (6 months) was defined as the absence of signs and symptoms of VL (no fever, reduced spleen size, hematological parameters recovered, and weight gained) and having not required any rescue treatment during the trial.

### Statistical Analyses

The minimal sample size was based on the pharmacokinetic clinical trial simulations for the primary pharmacokinetic endpoint, using the method of Dorlo and colleagues [18]. Including potential non-compliance, this provided a trial sample of 30 patients. For the primary pharmacokinetic endpoint, plasma miltefosine concentrations were measured in all patients receiving at least 1 miltefosine dose. All patients who were administered the first dose of miltefosine constituted the safety population. The primary population for efficacy analysis at days 28 and 210 was the intention-to-treat population (ITT). The per-protocol (PP) population included patients with no pre-specified major protocol deviations relating to treatment compliance and baseline exclusion criteria.

Descriptive analyses were used for patient characteristics and biological data. Categorical variables were summarized using proportions. Continuous variables are presented as means (standard deviation) and medians (interquartile range). Box plots are used to present laboratory parameters and spleen sizes. The time to relief of fever was analyzed using a Kaplan-Meier curve. For efficacy analyses, the primary analysis population at days 28 and 210 was the ITT population. Furthermore, a PP analysis was also performed. The STATA version 13.1 program [19] was used for data analysis.

## RESULTS

### Patients

**Figure 1.**
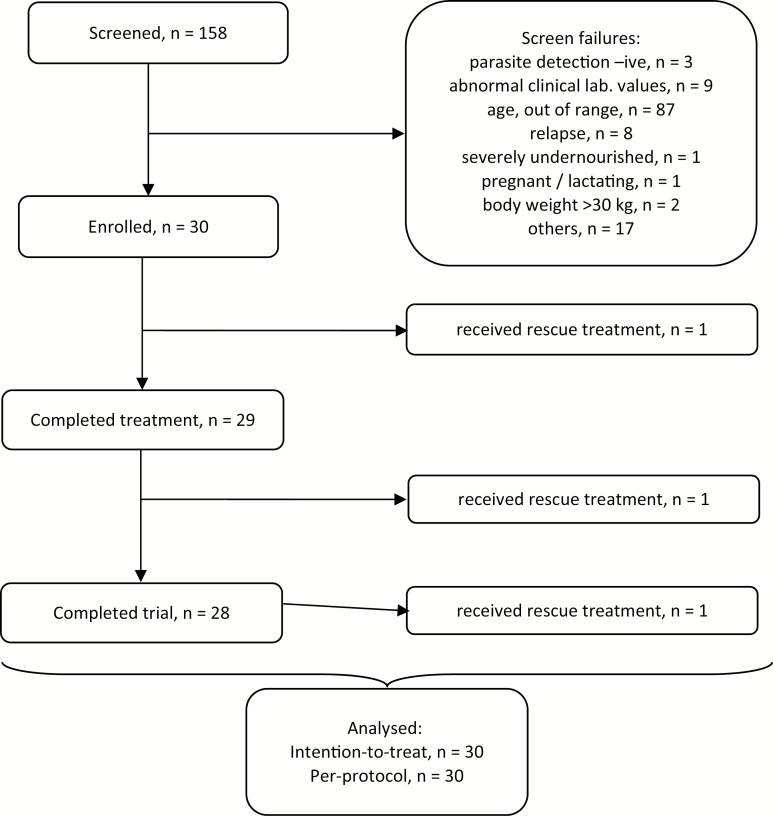
Disposition of patients.

Out of 158 suspected VL cases, 30 subjects were enrolled; their disposition during the trial is shown in Figure 1. Patients were excluded for various reasons, but mostly (87/158) because of age. Of those enrolled, 3 required rescue treatment: 1 during miltefosine treatment and 2 during the follow-up. The trial was completed without any loss to follow‐up. The ITT and PP populations were of identical sizes (n = 30).

The age distribution of the children was evenly spread between 4 and 12 years (Table 1). All children had fever and most had abdominal swelling (96.7%), mucosal pallor (96.7%), and splenomegaly (93%). Most were of normal weight, with 36.7% underweight and none overweight, but 63.3% had lost weight since the disease onset, with 53.3% having muscle wasting and 26.7% having appetite loss.

**Table 1.** Patients’ Baseline Characteristics

**Demographics**	**n (%**)	
Number of patients	30 (100)	
Aged 4–7 years	16 (53.3)	
Aged 8–12 years	14 (46.7)	
Females/males	8 (26.7)/22 (73.3)	
	**Mean ± SD**	**Median, IQR**
Age (years)	7.7 ± 2.1	7.0, 6.0–9.0
Weight (kg)	21.7 ± 4.2	21.8, 19.0–26.0
**Nutritional status** ^a^	n (%)	
Underweight	11 (36.7)	
Normal	19 (63.3)	
Overweight	0 (0.0)	
**Vital signs**	**Mean ± SD**	**Median, IQR**
Heart rate (beats/min)	113.9 ± 18.8	113.5, 100.0–122.0
Systolic BP (mm Hg)	97.8 ± 8.9	97.5, 89.0–106.0
Diastolic BP (mm Hg)	59.9 ± 9.3	59.5, 53.0–66.0
Temperature (°C)	37.0 ± 1.3	36.6, 36.1–37.9
**Signs and symptoms** (referred by the patient)	n (%)	
Fever	30 (100.0)	
Abdominal swelling	29 (96.7)	
Loss of appetite	8 (26.7)	
Weight loss	19 (63.3)	
Diarrhea	0 (0.0)	
Coughing	14 (46.7)	
Epistaxis	7 (23.3)	
Other bleeding signs	0 (0.0)	
Jaundice	1 (3.3)	
**Clinical characteristics** (per physical examination)	n (%)	
Mucosal pallor	29 (96.7)	
Cervical lymphadenopathy	4 (13.3)	
Jaundice	0 (0.0)	
Axillary lymphadenopathy	2 (6.7)	
Inguinal lymphadenopathy	1 (3.3)	
Muscle wasting	16 (53.3)	
Post–Kala-azar dermal leishmaniasis	0 (0.0)	
Other significant systemic characteristics	0 (0.0)	
	**Mean ± SD**	
Spleen size (cm)	9.1 ± 4.3	
Liver size (cm)	2.6 ± 2.0	
**Hematology** ^b^	**Mean ± SD**	**Median, IQR**
Hemoglobin (g/dL)	7.0 ± 1.1	6.9, 6.1–8.0
Hematocrit (%)	23.0 ± 3.9	22.9, 19.1–26.5
White‐cell count (x10^3^/µL)	2.7 ± 1.2	2.7, 1.8–3.0
Neutrophils (%)	36.1 ± 11.6	37.0, 28.0–43.0
Lymphocytes (%)	58.2 ± 10.5	60.0, 49.0–64.0
Monocytes (%)	5.7 ± 4.1	6.0, 2.0–9.0
Eosinophils (%)	0.03 ± 0.18	-
Platelets (x10^3^/µL)	128.4 ± 82.7	107.0, 83.0–139.0
**Clinical chemistry** ^c^	**Mean ± SD**	**Median, IQR**
Aspartate aminotransferase (U/L)	48.7 ± 36.2	35.0, 29.0–50.0
Alanine aminotransferase (U/L)	26.6 ± 18.6	20.5, 14.0–36.0
Total bilirubin (μmol/L)	5.7 ± 3.1	5.0, 3.0–6.8
Creatinine (μmol/L)	47.4 ± 8.4	47.5, 41.0 to 53.0

Abbreviations: BP, blood pressure; IQR, interquartile range; SD, standard deviation; WHO, World Health Organization.

^a^Based on WHO standardized nutritional status. Children with severe malnutrition were excluded (z‐score <−3). For children aged <5 years, weight for height was used: children were considered underweight when their z‐score <−2 and overweight when their z‐score >2. For children aged 5–12 years, body mass index for age was used: children were considered underweight when their z‐score <−2 and overweight when their z‐score >1.

^b^The following reference ranges were used for hematology parameters: hemoglobin (10–14.5 g/dL), white blood cell count (4–10 x10^3^/µL), neutrophils (42–75%), and platelets (120–400 x10^3^/µL).

^c^The following reference ranges were used for clinical chemistry parameters: aspartate aminotransferase (22–60 U/L), alanine aminotransferase (12–45 U/L), total bilirubin (<17 µmol/L), and creatinine (17.6–88 µmol/L)

### Pharmacokinetics

The patients received a median daily allometric miltefosine dose of 3.2 mg/kg/day (range, 2.7–3.9 mg/kg/day). The quantitative analysis of plasma miltefosine met standard Food and Drug Administration criteria, with accuracies and precisions within ±15% and ≤15%, respectively. Excluding pre-treatment samples, which were all below the lowest level of quantification, as expected, a total of 206 samples were collected from 30 patients, as per protocol. We excluded 3 samples from the data analysis, since a steep (>70%) decrease in miltefosine concentration was observed during treatment, which was physiologically improbable due to the long elimination half-life of miltefosine [18].

### Observed Miltefosine Exposure After Allometric Dosing

**Figure 2.**
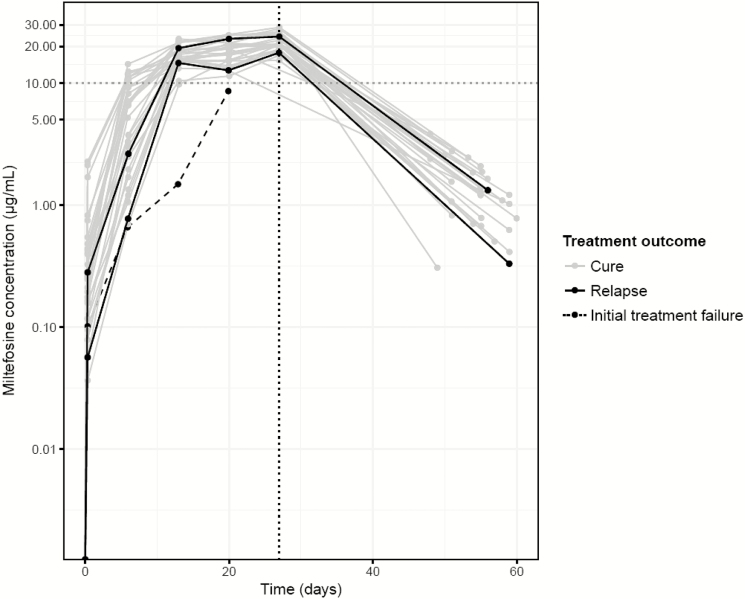
Individual plasma miltefosine concentration-time profiles: the gray lines indicate the patients who were cured of visceral leishmaniasis, the full black lines indicate the 2 patients who required rescue treatment during follow-up, and the dashed black line indicates the patient who initially failed on treatment. The vertical dotted line shows the end of treatment (day 28).

Miltefosine plasma concentration-time profiles after allometric dosing are depicted in Figure 2. Of the 3 patients who experienced treatment failure or relapse, 2 had substantially lower miltefosine accumulations than the cured patients (Figure 2). Unexpectedly, for 37% of patients, the miltefosine concentrations plateaued or even decreased between days 14 and 21 (change in concentration between -19% and +10%), after which concentrations increased >18% (range 18–58%) at day 28.

### Descriptive Comparison of Allometric Miltefosine Dosing with Historical, Conventional-dosing Data

Miltefosine exposure in the current allometric study was compared with pediatric data for conventional linear dosing (from the LEAP 0208 trial [11]). Due to differences in age limits for inclusion, the LEAP 0208 cohort (n = 21) had a higher median age (10 years, range 7–12 years) and a higher median body weight (24 kg, range 16–34 kg) [11]. As there was no difference in exposure between the 4–6 year and 7–12 year age groups in the present study, all patients with available pharmacokinetics (PK) data were included in this comparison with the LEAP 0208 trial.

Although not statistically significant, children receiving allometric dosing showed a trend (*P* = .07) for much more rapid miltefosine accumulation than children on conventional dosing, with median day-7 plasma concentrations of 5.88 µg/mL (range 0.66–14.3 µg/mL) versus 2.67 µg/mL (range 0.70–12.8), respectively. However, the median total exposure was only slightly increased with allometric dosing, resulting in a 6% and 8% higher C_max_ and AUC_0-210_, respectively (Table 2). For comparison, the observed total exposure after allometric dosing was still lower than that in adults receiving the conventional linear dosing (median non-compartmental analysis AUC_0-210_ 582 versus 836 µg∙day/mL, respectively; data not published). The variability (coefficient of variation%) of miltefosine C_max_ values was 2-fold lower with allometric dosing (15.7%) than with conventional dosing (30.5%) when comparing the pediatric populations. Similarly, AUC_0-210_ values were less variable with allometric dosing than with conventional dosing (Table 2). Furthermore, the proportion of patients with a C_max_ lower than the target of 17.9 µg/mL was 14.8% in patients treated with allometric dosing, as compared to 28.6% in conventional dosing (Table 2).

### Safety

Table 3 summarizes the frequency of AEs recorded during the trial and Table 4 lists the AEs according to the Medical Dictionary for regulatory activities (MedDRA) categorization system (System Organ Class/Preferred Term), their relationship to the study drug, and severity (as per CTCAE v4.0). A total of 110 treatment-emergent adverse events (TEAEs) were reported in 30 subjects. All patients had at least 1 TEAE, with the most frequent being anaemia, neutropenia, malaria, and upper respiratory infection. There were 13 patients (43%) that presented a total of 19 TEAEs related to the study drug, which are referred to as treatment-emerging adverse drug reactions (TEADRs). Among these, the most common were neutropenia and vomiting, which were reported in 20% and 17% of patients, respectively. No patient discontinued treatment due to an AE, and cases of vomiting were not associated with treatment compliance failure. There were 5 (4.5%) Grade 4 TEAEs reported (1 anaemia, 4 neutropenia). The Grade 4 neutropenia cases (<500/mm^3^) occurred in subjects with Grade 3 low neutrophil counts at baseline that worsened in severity after the treatment initiation. The majority of neutropenia cases occurred during the first days of treatment, were temporary and asymptomatic, resolved spontaneously, and did not require any intervention. Only mild (Grade 1 CTCAE) increases in aspartate aminotransferase and alanine aminotransferase levels were observed. Creatinine levels remained stable for all subjects during the treatment period, and only 1 Grade 2 increase was observed. These Grade 1 and 2 increases were not considered clinically significant and not reported as AEs by the investigator.

**Table 3.** Frequency of Adverse Events

Patients enrolled and receiving at least 1 dose, number (%)	30 (100)
Patients with at least 1 AE, serious or not, number (%)	30 (100)
Patients with adverse drug reaction, number (%)	13 (43)
Patients with an AE not related to the study drug, number (%)	30 (100)
Patients with at least 1 serious AE, number (%)	2 (7)
TEADR, number	19
TEADR per patient, median (range)	1 (1–6)
Patients whose treatment was stopped due to a TEAE, number (%)	0 (0)
Patients experiencing ≥1 episode of repeated vomiting, number (%)	0 (0)
Patients experiencing ≥1 CTC grade 3 or 4 TEADR, number (%)	5 (17)

Abbreviations: AE, adverse event; CTC, Common Terminology Criteria; TEAE, treatment-emergent adverse events; TEADR, treatment-emerging adverse drug reaction.

**Table 4.** List of Treatment-Emergent Adverse Events by Relatedness to Study Drug and Severity

System Organ Class	Preferred MedDRa Term	Total	Study Drug Relatedness		Grade of Severity			
			Not Related	Related	1	2	3	4
		Number of TEAEs						
Blood and lymphatic system disorders	Anaemia	14	13	1	0	1	12	1
	Leukopenia	5	5	0	0	0	5	0
	Neutropenia	1	7	7	0	1	9	4
	Thrombocytopenia	3	3	0	0	0	3	0
Gastrointestinal disorders	Constipation	1	1	0	1	0	0	0
	Diarrhea	3	1	2	3	0	0	0
	Hyperacidity	1	1	0	1	0	0	0
	Nausea	1	0	1	1	0	0	0
	Rectal prolapse	1	0	1	1	0	0	0
	Vomiting	6	1	5	6	0	0	0
General disorders and administration site conditions	Hypothermia	1	1	0	0	1	0	0
	Puncture site pain	1	1	0	1	0	0	0
	Pyrexia	6	5	1	1	3	2	0
Infections and infestations	Abscess	2	2	0	0	2	0	0
	Ascariasis	1	1	0	0	1	0	0
	Bronchopneumonia	1	1	0	0	1	0	0
	Conjunctivitis	1	1	0	1	0	0	0
	Fungal infection	1	1	0	1	0	0	0
	Herpes simplex	1	1	0	1	0	0	0
	Lower respiratory tract infection	2	2	0	1	1	0	0
	Malaria	12	12	0	6	6	0	0
	Otitis media	3	3	0	0	3	0	0
	Tinea capitis	4	4	0	2	2	0	0
	Tonsillitis	1	1	0	0	1	0	0
	Upper respiratory infection	12	12	0	9	3	0	0
	Varicella	1	1	0	0	1	0	0
	Wound infection	1	1	0	0	1	0	0
Injury, poisoning, and procedural complications	Transfusion Reaction	1	1	0	0	1	0	0
Investigations	Blood bilirubin unconjugated increased	1	0	1	0	1	0	0
Nervous system disorders	Headache	1	1	0	0	1	0	0
Respiratory, thoracic, and mediastinal disorders	Cough	3	3	0	3	0	0	0
	Epistaxis	2	2	0	1	1	0	0
	Oropharyngeal pain	1	1	0	1	0	0	0
Skin and subcutaneous tissue disorders	Rash	1	1	0	0	1	0	0

Abbreviation: TEAEs, treatment-emergent adverse events.

There were 2 patients that each had a SAE. The first had a case of “transfusion reaction” that was considered an important medical event by the investigator, occurring on day 203 after the treatment start. The other case was life-threatening anaemia (PT), occurring on day 19. This patient was later assessed as an initial failure. Both SAEs were reported as unrelated to miltefosine treatment by the investigator, and the patients fully recovered.

### Efficacy

Each enrolled patient belonged to both the PP and ITT populations. The 28-day and 210-day cure rates were 96.7% (95% confidence interval, 83-100%; 29/30 patients) and 90% (95% confidence interval, 74-98%; 27/30 patients), respectively. There were 3 patients that received Ambisome rescue treatment: 1 at day 25 (initial failure), 1 at day 168 (relapse), and 1 at day 206 (relapse).

**Figure 3.**
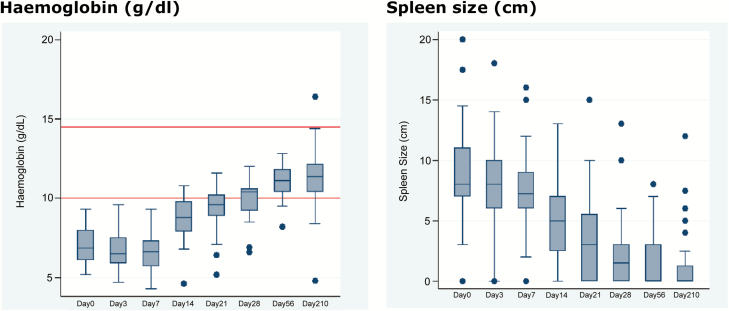
Box plots of selected efficacy clinical parameters during treatment (day 0 to day 28) and follow-up. The box plots represent the interquartile ranges, the whiskers represent minimum and maximum values, and the dots outside the whiskers are outlier values. Red lines in the hemoglobin figure represent the lower and upper limits of normal.

The clinical response was observed in several parameters, including spleen and liver sizes, which decreased progressively during the treatment and follow-up period (up to day 210; Figure 3). Hemoglobin and hematocrit increased towards normal levels by the end of treatment and remained normal during follow-up (Figure 3). Fever was cleared in nearly all patients by day 14 of treatment, and in all patients by the end of treatment (see Supplementary Figure S1). Weight gain was observed for all patients, with an 8% average increase at the end of the study compared to baseline.

## DISCUSSION

The present study is the first to assess the pharmacokinetics, safety, and efficacy profile of miltefosine in pediatric VL patients treated with an allometric dosing regimen, which provided a 28% higher median daily dose than conventional linear dosing. Our pharmacokinetic data indicate more rapid accumulation after allometric dosing in the first weeks of treatment, with both C_max_ and AUC_0-210_ being only slightly increased (6% and 8%, respectively), probably due to a plateau in accumulation in the third week of treatment, implying a non-linear dose proportionality of miltefosine pharmacokinetics (see online Supplementary Materials).

Variability in exposure decreased almost 2-fold with the allometric dosing compared to the conventional dosing (as shown by the spread of C_max_ and of AUC_0-210_; Table 2). The low variability observed in miltefosine concentrations between patients can also be seen in Figure 2. These data indicate that allometric dosing of miltefosine allows a more consistent systemic exposure to the drug treatment than linear dosing. The individual patients’ C_max_ values from the non-compartmental pharmacokinetic analyses (Table 2) also indicate that fewer children (15%) on allometric dosing than children (29%) on linear dosing had plasma miltefosine levels below the threshold of 17.9 μg/mL, which has been previously shown to be related to a higher probability of disease relapse [21]. Additionally, miltefosine concentrations in the first week of treatment were highly increased after allometric dosing, as compared to linear dosing, when comparing the pediatric populations (data not shown). This may be pivotal, both in terms of efficacy as well as driving the emergence of drug resistance, given that the parasite biomass is highest at this stage of treatment.

**Table 2.** Comparison of Noncompartmental Pharmacokinetic Parameters After Conventional Linear and Allometric Dosing in Separate Groups of Miltefosine-treated Eastern African Pediatric Patients With Visceral Leishmaniasis

	Conventional Dosing			Allometric Dosing		
Number of patients	21			27		
Demographics	LEAP0208			LEAP0714^a^		
Age in years, median (range)	10 (7–12)			7 (4–12)		
Weight in kg, median (range)	24 (16–34)			22 (13–30)		
Height in meters, median (range)	1.35 (1.07–1.53)			1.25 (0.99–1.45)		
Gender, % female	24%			27%		
Pharmacokinetic parameters						
	Median	Range	RSD	Median	Range	RSD
AUC_0-210_, μg*day/mL	539	295–1110	35.3%	582	392–817	18.9%
C_max_,μg/mL	19.9	14.4–37.7	30.5%	21.0	15.5–28.6	15.7%
C_max_ < 17.9 µg/mL target, n (%)	6/21 (28.6%)			4/27 (14.8%)		

Data are from LEAP 0208 and LEAP 0714 Trials: 1 outlier data point was excluded from analyses of each cohort.

Abbreviations: AUC_0-210_, area under the curve; C_max_, plasma maximum concentration; RSD, relative standard deviation.

^a^Data from 3 patients were excluded: 2 patients had an anomalous decline in miltefosine values between days 21 and 28 and 1 patient had no values after day 21.

The improved miltefosine pharmacokinetics could at least partially explain the much improved 6-month cure rate (90%), compared to the rate we previously reported in Eastern African children (59%, LEAP 0208) [11]. In fact, the efficacy observed in children treated with allometric dosing was similar to that observed in adults (86%) treated with a 28-day conventional treatment regimen [11], despite the lower average drug exposure compared to adults.

The present non-compartmental analysis is limited by the sparse sampling and the observed pharmacokinetic non-linearity, which may, for example, cause an underestimation of exposure during treatment and an overestimation during follow-up. We are currently developing a model-based analysis of pharmacokinetic data pooled from several trials, to characterize the observed non-linearities in pediatric VL patients in Eastern Africa.

Our findings are an important basis for the further development of miltefosine as an effective oral medicine for use in combination treatment of VL in Eastern African children. We show that allometric miltefosine dosing in children is safe and more effective than conventional dosing. Further development of miltefosine should adopt allometric dosing in patients weighing ˂30 kg. A phase III trial is envisaged to assess combining an allometric regimen of miltefosine with paromomycin in VL patients in Eastern Africa, as compared to sodium stibogluconate and paromomycin. Positive results would provide a basis for the provision of an alternative treatment that is more patient friendly and requires shorter hospitalizations than the current standard treatment, by replacing the much more toxic sodium stibogluconate with oral miltefosine. Miltefosine allometric dosing would be of potential benefit beyond Eastern Africa, as there is also an urgent need in South America and Asia for more tolerable and more convenient oral treatments for children affected by cutaneous leishmaniasis and post–kala-azar dermal leishmaniasis.

## Supplementary Data

Supplementary materials are available at *Clinical Infectious Diseases* online. Consisting of data provided by the authors to benefit the reader, the posted materials are not copyedited and are the sole responsibility of the authors, so questions or comments should be addressed to the corresponding author.

Supplementary Material

## Notes

***Acknowledgments.*** The authors thank all members of the field teams at the study sites of Kacheliba Hospital and Amudat Hospital, including clinicians, nurses and laboratory technicians, clinical monitors, and the Data Safety Monitoring Board, for their contributions to the study. They sincerely thank the visceral leishmaniasis patients and the parents of the pediatric patients for their willingness to be enrolled in this study and their cooperation. They also thank the local communities and acknowledge the Ministries of Health of Kenya and Uganda for their support, and the County Government of Kacheliba for allowing them to undertake the study at Kacheliba hospital. They thank Gerard McGregor, OmniScience SA, Geneva, who provided medical writing services on behalf of Drugs for Neglected Diseases Initiative (DNDi), and Susan Wells and Byron Arana for proof-reading the manuscript.

***Disclaimer.*** This manuscript is published with permission from the Director of Kenya Medical Research Institute.

***Financial support.*** This work was supported through DNDi by the European Union Seventh Framework Programme Africoleish (grant number 305178); the World Health Organization - Special Programme for Research and Training in Tropical Diseases (WHO-TDR); the French Development Agency, France (grant number CZZ2062); Department for International Development, UK; the Federal Ministry of Education and Research through KfW, Germany; the Medicor Foundation, Liechtenstein; Médecins Sans Frontières, International; the Swiss Agency for Development and Cooperation, Switzerland (grant number 81017718); and the Dutch Ministry of Foreign Affairs, The Netherlands (grant number PDP15CH21).

***Potential conflicts of interest.*** All authors: No reported conflicts of interest. All authors have submitted the ICMJE Form for Disclosure of Potential Conflicts of Interest. Conflicts that the editors consider relevant to the content of the manuscript have been disclosed.
