# Supplementary material for: Pharmacokinetics, Safety, and Efficacy of an Allometric Miltefosine Regimen for the Treatment of Visceral Leishmaniasis in Eastern African Children: An Open-label, Phase II Clinical Trial
Source: Clin Infect Dis. 2018 Sep 5;68(9):1530–8. doi: 10.1093/cid/ciy747 (PMC6481997; doi:10.1093/cid/ciy747)
Supplement: Supplementary Material [file ciy747_suppl_supplementary_material.docx]

**LEAP7014 trial patient inclusion and exclusion criteria**

Each recruited patient fulfilled the following inclusion criteria: (i) clinical signs and symptoms of VL and confirmatory parasitological microscopic diagnosis, (ii) aged ≥4 years and ≤12 years, (iii) able to comply with the study protocol, (iv) written informed consent signed by parents(s) or legal guardian, and children`s assent (>8-12 years, in Uganda only), (v) body weight of <30 kg. They each had none of the following exclusion criteria: (i) relapsed VL, (ii) received any anti-leishmanial drugs within the previous six months, (iii) severe malnutrition (= a z score of <-3 on weight-for-height WHO reference curves, if aged <5 years or a z score of <-3 on BMI-for-age WHO reference curves, if aged 5-12 years), (iv) positive HIV diagnosis, (v) previous history of hypersensitivity to miltefosine, (vi) concomitant severe infection or any other serious underlying disease, (vii) any condition associated with splenomegaly, such as schistosomiasis, (viii) had reached menarche (female patients), (ix) haemoglobin concentrations of <5 g/dL, (x) white blood cell count of <1x10^3^/mL blood, (xi) platelet count of <40,000/mL blood, (xii) abnormal liver function (= blood alanine-transferase and aspartate transferase levels >3-times above upper limit of normal range), (xiii) blood bilirubin levels >1.5-times greater than upper limit of normal range, (xiv) serum creatinine levels above upper limit of normal range, (xv) clinical signs of severe VL (jaundice and bleeding), (xvi) unable to comply with study protocol.

**Daily Miltefosine allometric dosing table for female and male paediatric subjects adapted from Dorlo et al (2012)**

**Plasma miltefosine bioanalysis**

Plasma samples were stored and transported at maximally -20^o^C under temperature monitoring condition to the Bioanalytical Laboratory (at Antoni van Leeuwenhoek Hospital, Netherlands Cancer Institute, Amsterdam, the Netherland) where they underwent solid-phase extraction of the miltefosine content, followed by chromatographic separation and quantification using liquid chromatography coupled to tandem mass spectrometry (LC-MS/MS), following a previously developed and validated method [1]. The lower limit of quantitation (LLOQ) of the assay was 4 ng/mL and the upper limit of quantitation was 1,000 ng/mL, with all accuracies and precisions within <15% deviation.

**Comment on “plateau” in miltefosine pharmacokinetics between days 14-21**

The more rapid early accumulation of miltefosine after allometric dosing without significantly increasing C_max_ and AUC_0-210_ may be due to the observed plateau in accumulation in the third week of treatment. This “plateau” indicates an unexpected non-linearity in the miltefosine pharmacokinetics due to an unknown saturable process that transiently decreases bioavailability. In the LEAP 0208 trial, day 21 plasma miltefosine concentrations were not measured in the children but were in the adult patients and a ‘plateau’ in the miltefosine pharmacokinetics was not observed [2]. A number of factors were explored such as levels of albumin, co-infections, concomitant medications used before and during treatment, but none could explain the presence of this ‘plateau’ in the sub-set of patients. Treatments were administered under observation ensuring 100% compliance, so this cannot explain as well this unusual pharmacokinetic profile.

References:

[1] Dorlo TP, Hillebrand MJ, Rosing H, Eggelte TA, de Vries PJ, Beijnen JH. Development and validation of a quantitative assay for the measurement of miltefosine in human plasma by liquid chromatography-tandem mass spectrometry. J Chromatogr B Analyt Technol Biomed Life Sci **2008**; 865(1-2): 55-62.

[2] Wasunna M, Njenga S, Balasegaram M, et al. Efficacy and Safety of AmBisome in Combination with Sodium Stibogluconate or Miltefosine and Miltefosine Monotherapy for African Visceral Leishmaniasis: Phase II Randomized Trial. PLoS Negl Trop Dis 2016a; 10(9): e0004880.

**Figure S1: Efficacy: Time to relief of fever (days after treatment onset)**
